# Syndrome of inappropriate ADH secretion (SIADH) associated with citalopram use

**DOI:** 10.4103/0019-5545.55960

**Published:** 2005

**Authors:** Vivek C. Kirpekar, Prashant P. Joshi

**Affiliations:** * Consultant Psychiatrist, Kirpekar Hospital, Nagpur; ** Associate Professor, in Medicine Government Medical College, Nagpur

**Keywords:** SIADH, citalopram, hyponatraemia

## Abstract

Selective serotonin reuptake inhibitors (SSRIs) can cause the syndrome of inappropriate antidiuretic hormone secretion (SIADH). SIADH is associated with hyponatraemia without oedema. We report the case of a patient who developed acute-onset hyponatraemia that progressed rapidly to serious neurological dysfunction shortly after the introduction of citalopram. All SSRIs including citalopram should be used with care in the elderly. The water and electrolyte balance should be monitored carefully during SSRI therapy.

## INTRODUCTION

Selective serotonin reuptake inhibitors (SSRIs) were developed to formulate reuptake-blocking antidepressant drugs that lacked the troublesome side-effects associated with blockade of other neurotransmitter systems. The convenience of once-a-day dosing and mild side-effects have led to extensive use of these agents.^1^

The syndrome of inappropriate antidiuretic hormone secretion (SIADH) is associated with hyponatraemia without oedema,^2^ caused either by stimulating the release of vasopressin or by potentiating its action on the renal tubules. The symptoms of SIADH include lethargy, headache, insomnia, nervousness, apathy, agitation, confusion, convulsions and coma; and these are decided by the rate of fall in serum sodium concentration. Patients who have hyponatraemia and concentrated urine (osmolality >300 mOsm/kg) should be suspected to have SIADH. The diagnosis is supported by the finding of low normal or subnormal levels of blood urea nitrogen, serum uric acid, creatinine and albumin.^3^ Diuretic therapy, tumours, hypothyroidism, respiratory and central nervous system diseases are common aetiological factors for SIADH.

Many drugs including tricyclic antidepressants, phenothiazines, carbamazepine, narcotics and all SSRIs can cause SIADH. Antidepressant-induced SIADH has been reported mostly in patients above 65 years of age, mostly due to the use of fluoxetine.^1^,^3^,^4^

Citalopram, one of the latest SSRIs, was introduced in the Indian market in October 2001 and, so far, SIADH associated with citalopram use has not been reported from India.

## THE CASE

A 78-year-old man suffering from major depression was put on 10 mg of Tab. citalopram daily from 10 October 2003. All relevant investigations including serum electrolyte levels were normal. In the past, he was asymptomatic and had not received any medication.

On day 4 of treatment, he complained of anorexia, nausea and dysphagia. His condition rapidly deteriorated over the next 3 days. He developed tremors, a mask-like face, truncal weakness and sustained repeated falls. His sensorium was altered, and he became disoriented, restless and agitated. Citalopram was stopped.

A provisional diagnosis of a brainstem infarct was ruled out by the normal MRI scan. He was admitted to the intensive care unit on 18 October 2003. He had no oedema and had a blood pressure of 150/80 mmHg. Examination of the chest and cardiovascular system was normal. His serum sodium was 107 mmol/L and serum potassium 3.9 mmol/L. The electrocardiogram revealed a normal sinus rhythm, a heart rate of 66/min, no ST-T changes and a few ventricular ectopics. His blood urea was 18.9 mg/dl and serum creatinine 0.79 mg/dl. His urinary sodium was 166 mmol/L and urinary osmolality by the freezing point method was 332 mOsm/kg. The serum proteins were 5 g/dl (total) and 2.9 g/dl (albumin).

A diagnosis of hyponatraemia due to SIADH was made. He was treated with 3% normal saline infusion at the rate of 20–25 ml/hour. Over the next 72 hours his sensorium improved and he was discharged after his serum sodium levels increased to 127 mmol/L. He was maintained on water restriction (one-and-a-half litre/day) and oral salt supplementation. It took almost 3 weeks for his serum sodium levels to rise to 133 mmol/L.

## DISCUSSION

Hyponatraemia (serum sodium <135 mmol/L) is an increasingly recognized adverse effect of SSRIs and venlafaxine.^1^,^4^,^6^ However, there are few case reports of citalopram-associated SIADH.

A recent overview of data on the safety of citalopram from clinical trials, published clinical reports and case reports does not mention hyponatraemia even in the elderly population.^7^

Fisher *et al*.^8^ reported the case of a 92-year-old woman who developed severe hyponatraemia with deep coma, seizures, atrial fibrillation and muscle damage after only two doses of citalopram. They also reviewed 14 previously published and 28 spontaneously reported cases. Christe and Vogt^9^ reported 5 cases of severe hyponatraemia (<125 mmol/L) associated with citalopram use, which developed on the third day of administration of the drug. Following discontinuation of SSRI and start of fluid restriction, the hyponatraemia resolved. A few single case reports have been published of elderly patients developing SIADH after citalopram use in therapeutic doses.^2^,^10^ The patients improved with discontinuation of citalopram.

Shortly after the introduction of citalopram, our patient developed acute-onset hyponatraemia that progressed rapidly to serious neurological dysfunction. The laboratory findings of a low serum sodium level along with high urinary osmolality and high urinary sodium helped to establish the diagnosis of SIADH. Normal electrolyte levels before the initiation of citalopram, absence of other aetiological factors of hyponatraemia and its correction after stopping citalopram, suggest a cause-and-effect relationship.

## CONCLUSION

Citalopram should be used with care in the elderly. The water and electrolyte balance should be monitored carefully during SSRI therapy.

## References

[1] Marangell LB, Silver JM, Goff DC, Hales RE, Yudofsky SG (2003). Pharmacotherapy and electroconvulsive therapy. Textbook of clinical psychiatry.

[2] Bourgeois JA, Babine SE, Bahadur N (2002). A case of SIADH and hyponatremia associated with citalopram. Psychosomatics.

[3] Robertson GL, Braunwald E, Fauci A, Kasper D (2001). Disorders of neurohypophysis. Harrison's principles of internal medicine.

[4] Kirbi D, Harrigan S, Ames D (2002). Hyponatremia in elderly psychiatric patients treated with selective serotonin reuptake inhibitors and venlafaxine: A retrospective controlled study in an inpatient unit. Int J Geriatr Psychiatry.

[5] Liu BA, Mittmann N, Knowles SR (1996). Hyponatremia and the syndrome of inappropriate secretion of antidiuretic hormone associated with the use of selective serotonin reuptake inhibitors: A review of spontaneous reports. Can Med Assoc J.

[6] Bouman WP, Pinner G, Johnson H (1998). Incidence of selective serotonin reuptake inhibitor (SSRI) induced hyponatremia due to the syndrome of inappropriate antidiuretic hormone (SIADH) secretion in the elderly. Int J Geriatr Psychiatry.

[7] Nemeroff CB (2003). Overview of the safety of citalopram. Psychopharmacol Bull.

[8] Fisher A, Davis M, Croft-Baker J (2002). Citalopram-induced severe hyponatremia with coma and seizure. Case report with literature and spontaneous report review. Adverse Drug React Toxicol Rev.

[9] Christe C, Vogt N (1999). SSRI-induced SIADH in older people. Am Geriatr Soc.

[10] Barclay TS, Lee AJ (2002). Citalopram-associated SIADH. Ann Pharmacother.

